# Preterm Birth: Pathophysiology, Prevention, Diagnosis, and Treatment

**DOI:** 10.1155/2015/417965

**Published:** 2015-08-30

**Authors:** Igor Hudić, Babill Stray-Pedersen, Vajdana Tomić

**Affiliations:** ^1^Clinic of Gynecology and Obstetrics, University Clinical Center, 75000 Tuzla, Bosnia and Herzegovina; ^2^Division of Obstetrics and Gynaecology, Rikshospitalet, Faculty of Medicine, University of Oslo, 0372 Oslo, Norway; ^3^Clinic for Gynecology and Obstetrics, University Hospital Mostar, 88000 Mostar, Bosnia and Herzegovina

*Preterm* delivery before 37 gestational weeks is a major challenge in perinatal health care. Over the past 30, the incidence of preterm delivery in most developed countries has been about 7–10% of live births. Some evidence shows that this incidence has increased slightly in the past few years, but the rate of birth before 32 weeks' gestation is almost unchanged, at 1-2%. Several factors have contributed to the overall rise in the incidence of* preterm* delivery. These factors include increased use of assisted reproduction techniques, increasing rates of multiple births, and more obstetric intervention. Progesterone is the key hormone maintaining pregnancy. Numerous progesterone effects can be demonstrated by laboratory studies involving every tissue of the reproductive tract, the myometrium, decidua, cervix, and fetal membranes. In particular, progesterone can alter the response to cytokines, inhibit prostaglandin and nitric oxide synthesis, reduce corticotrophin-releasing hormone (CRH) synthesis, block cervical stromal degradation, and induce cervical stromal matrix protein secretion. By altering both the mechanical and physiologic functions of the cervix, cervical performance may be substantially enhanced by these agents. Presumably, progesterone may alter the rate of cervical stromal degradation via altering secretion of matrix metalloproteases by diminishing prostaglandin and nitric oxide synthesis and minimizing neutrophil recruitment. The diverse aetiology of preterm delivery makes its prediction difficult. A substantial part of unexplained* preterm* deliveries might be attributable to a deleterious immune response of the mother toward the foetus. A growing body of evidence suggests that progesterone might play a significant role in establishing an adequate immune environment during the early stages of pregnancy. In the presence of progesterone, lymphocytes of pregnant women release a protein named the progesterone induced blocking factor (PIBF)^7^ which mediates the immunomodulatory and antiabortive effects of progesterone. Immunologic recognition of pregnancy and subsequent activation of maternal immune system result in an upregulation of progesterone receptors on activated lymphocytes among placental cells and CD8+ cells. In the presence of sufficient progesterone levels, these cells synthesize PIBF. Patients at risk of preterm delivery presented increased proinflammatory cytokines, low PIBF, and reduced IL-10 expressions on lymphocytes. PIBF alters the profile of cytokine secretion of activated lymphocytes shifting the balance toward Th_2_ dominance. During a normal uneventful pregnancy, the concentration of PIBF continuously increases from the 7th to the 37th gestational weeks. After the 41st week of pregnancy, PIBF concentrations dramatically decrease. In patients with a diagnosis of threatened premature labour, studies have shown that PIBF levels failed to increase during pregnancy. Identification of women with risk for preterm delivery would be a key for its prevention. No sufficiently specific marker, however, has so far been found. The diverse aetiology of preterm birth makes its prediction difficult. This special issue will give as some useful information but also questions which are important for our understanding of pathophysiology, prevention, diagnosis, and treatment of preterm birth.

*Igor  Hudić* *Babill  Stray-Pedersen* *Vajdana  Tomić*

